# Water and Sand Bags

**Published:** 1881-08

**Authors:** 


Water and Sand Bags.—These articles are among the most important of the domestic armamentaria of the sick room, and may be turned to most useful account in a large number of cases of disease. Now that local applications are attracting deserved attention in the treatment of many diseases, physicians would do well to direct the attention of families to the very neat and useful appliances, for using both warm and cold water, to be procured at almost any establishment in which rubber goods are sold. Of course, it would be a work of supererogation to attempt to instruct physicians in the methods of using hot sand bags, or hot and cold bags of water, in the management of their cases, or to enumerate the diseases in which they would be found valuable as remedies or adjuncts to other treatment. But all will be pleased with the convenience and ease with which *dry* heat and cold may be used in the elegant modern rubber bags arranged for those purposes.

